# Saline versus albumin fluid for extracorporeal removal with slow low-efficiency dialysis (SAFER-SLED): study protocol for a pilot trial

**DOI:** 10.1186/s40814-019-0460-3

**Published:** 2019-05-30

**Authors:** Edward G. Clark, Lauralyn McIntyre, Tim Ramsay, Alan Tinmouth, Greg Knoll, Pierre-Antoine Brown, Irene Watpool, Rebecca Porteous, Kaitlyn Montroy, Sophie Harris, Jennifer Kong, Swapnil Hiremath

**Affiliations:** 1 Division of Nephrology, Department of Medicine, The Ottawa Hospital, Riverside Campus, 1967 Riverside Drive, Ottawa, ON K1H 7W9 Canada; 2 Division of Critical Care, Department of Medicine, The Ottawa Hospital, 501 Smyth Road, Ottawa, ON K1H 8L6 Canada; 3 Ottawa Methods Centre, Ottawa Hospital Research Institute, 501 Smyth Road, Ottawa, ON K1H 8L6 Canada; 4 Division of Hematology, Department of Medicine, The Ottawa Hospital, 501 Smyth Road, Ottawa, ON K1H 8L6 Canada; 5 University of Ottawa, 75 Laurier Avenue, Ottawa, ON K1N 6N5 Canada

**Keywords:** Albumin, Saline fluid, Acute kidney injury, Dialysis, Low blood pressure, SLED treatment, Hypotension

## Abstract

**Background:**

Critically ill patients frequently develop acute kidney injury that necessitates renal replacement therapy (RRT). At some centers, critically ill patients who are hemodynamically unstable and require RRT are treated with slow low-efficiency dialysis (SLED). Unfortunately, hypotension is a frequent complication that occurs during SLED treatments and may limit the recovery of kidney function. Hypotension may also limit the amount of fluid that can be removed by ultrafiltration with SLED. Fluid overload can be exacerbated as a consequence, and fluid overload is associated with increased mortality.

Occasionally, intravenous albumin fluid is given to prevent or treat low blood pressure during SLED. The intent of doing so is to increase the colloid oncotic pressure in the circulation to draw in extravascular fluid, increase the blood pressure, and enable more aggressive fluid removal with ultrafiltration. Nonetheless, there is little evidence to support this practice and theoretical reasons why it may not be especially effective at augmenting fluid removal in critically ill patients. At the same time, albumin fluid is expensive.

As such, we present a protocol for a study to assess the feasibility of a randomized controlled trial evaluating the use of albumin fluid versus saline in critically ill patients receiving SLED.

**Methods:**

This study is a single-center, double-blind, and randomized controlled pilot trial with two parallel arms. It involves randomly assigning patients receiving SLED treatment in the ICU to receive either albumin (25%) boluses or normal saline fluid boluses (placebo) to prevent and treat low blood pressure.

**Discussion:**

The results of this pilot trial will help with planning a larger trial comparing the efficacy of the interventions in achieving fluid removal in critically ill patients with AKI on SLED. They will establish whether enough participants would participate in a larger study and accept the study procedures.

**Trial registration:**

This trial is registered on ClinicalTrials.gov Identifier NCT03665311, registered on September 11, 2018.

**Electronic supplementary material:**

The online version of this article (10.1186/s40814-019-0460-3) contains supplementary material, which is available to authorized users.

## Background

The incidence of acute kidney injury (AKI) treated with renal replacement therapy (RRT) in the ICU has more than quadrupled over the last two decades [1–3]. Given the diversity of acute and chronic co-morbidities in patients with RRT-requiring AKI [2], pharmacologic interventions are likely to remain elusive [4]. Nonetheless, efforts to optimize the administration of RRT hold promise for reducing mortality and maximizing the likelihood of renal recovery [5].

The clinical response to hemodynamic instability during slow low-efficiency dialysis (SLED) (or any form of RRT) is often to give intravenous fluid boluses (including albumin (25%)), reduce the ultrafiltration rate, or stop treatment altogether [6, 7]. All of these interventions lead to a more positive net fluid balance in patients with RRT-requiring AKI who usually have minimal urine output. It is unclear if giving fluids in this situation (often with the view to allow further ultrafiltration later on during the same treatment) is beneficial given that (a) simply reducing the ultrafiltration rate alone may lead to adequate time for vascular refilling from the extravascular compartment to improve hemodynamics [6–8] and (b) hemodynamic instability may be unrelated to ultrafiltration/preload reduction [9–11]. This is important given mounting evidence that greater fluid overload in this context is an independent risk factor for increased mortality and longer term dialysis dependence [12–18].

Our recent systematic review found that no studies have previously assessed the efficacy of giving albumin to prevent hypotension or augment fluid removal during SLED [19]. Albumin is frequently used during SLED treatment, and there is lack of data to suggest that albumin is more effective for potentiating fluid removal during SLED as compared with normal saline.

The primary aim of this pilot trial is to determine if, for critically ill patients treated with SLED for AKI, randomization to receive albumin (25%) boluses versus normal saline placebo boluses is feasible, with respect to the recruitment rate, blinding, and adherence to the protocol.

## Methods

### Study design and setting

The study design is a single-center, parallel design, double-blind, and randomized controlled trial comparing albumin boluses to normal saline boluses during SLED treatments. This study will take place in the mixed medical-surgical intensive care units of a large tertiary care hospital in Canada.

### Study population and timeline

The research coordinators will screen daily for patients that are in ICU to determine whether or not a patient is eligible for this pilot trial. This screening includes review of patient charts and discussion with nurses and physicians from the Circle of Care. The ICU is a closed unit in the sense that it is the same team recruiting for all studies in the unit. This pilot trial will require 24 months to complete. The participant recruitment and follow-up will take place during month 7 to 21 included as Table 1.

**Table 1:**
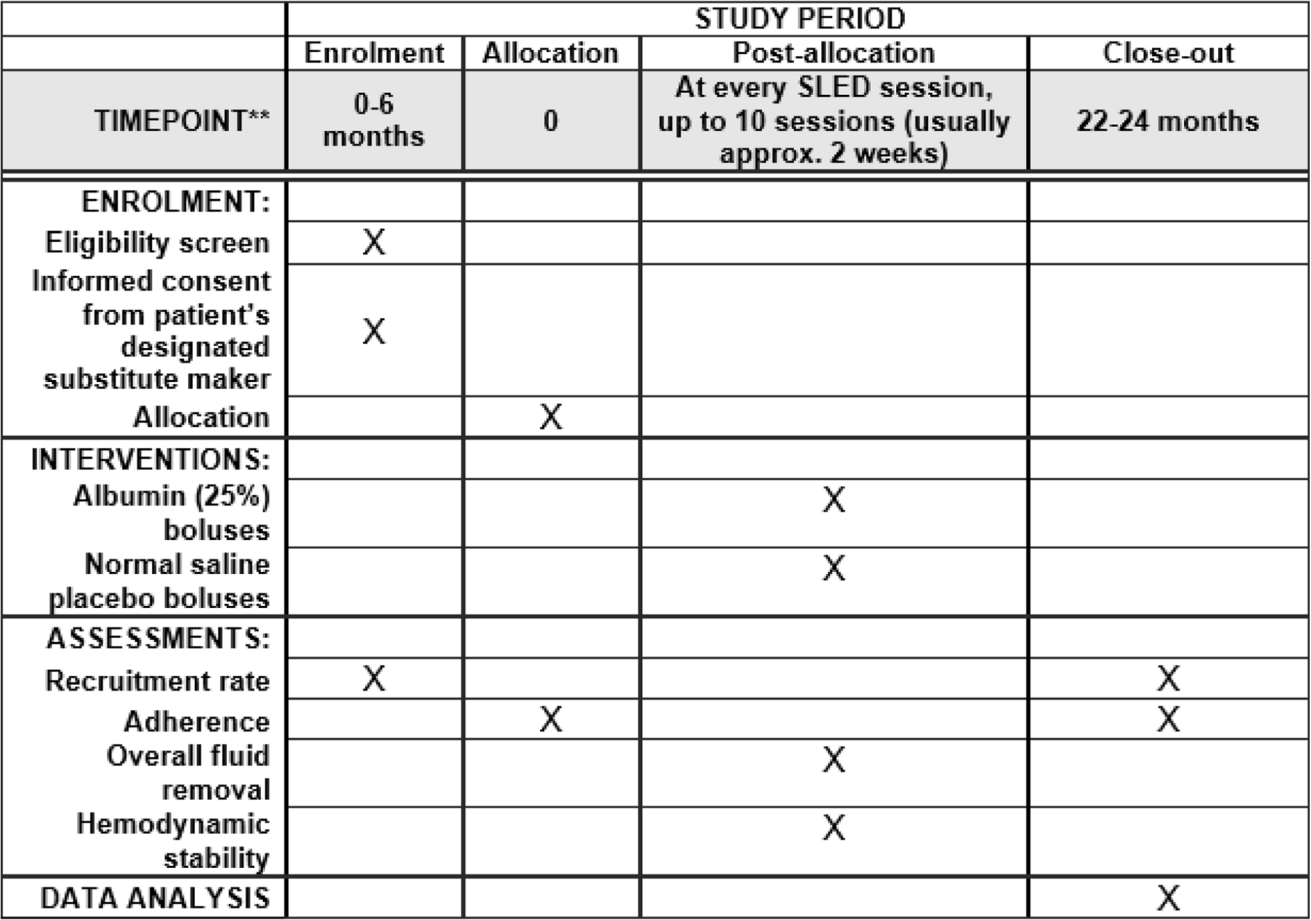
Study period content for the schedule of enrollment, interventions, and assessments for the SAFER-SLED pilot trial

Recommended content can be displayed using various schematic formats. See SPIRIT 2013 Explanation and Elaboration for examples from protocols

**List specific timepoints in this row

#### Inclusion criteria

- ≤ 18 years old
- Admission under the care of ICU and AKI treated with SLED (stage 3 AKI per Kidney Disease Improving Global Outcomes (KDIGO) AKI guidelines)

#### Exclusion criteria

- SLED initiated for non-AKI-related indication (such as concurrent intoxication; treatment of hypothermia)
- Receiving chronic dialysis treatments
- History of allergic reaction to albumin
- Pregnancy
- Patients with contraindications or known objections to blood transfusions

### Intervention

Participants will be randomized to receive either albumin (25%) boluses or normal saline placebo boluses during their SLED treatments in ICU (i.e., once randomized, the same fluid will be given during all subsequent SLED treatments for that patient). Since both interventions (albumin (25%) boluses and normal saline placebo boluses) will be administered by ICU nurses, we do not expect adherence to be a major issue. Nonetheless, we will monitor adherence on an ongoing basis as it is possible that the mid-treatment boluses are more likely to be missed if there is no hemodynamic instability observed during treatment. This pilot trial has no prohibitions in usual care, including physician-prescribed albumin or other fluids.

### Outcome measures

#### Feasibility

Recruitment rate, adherence to intervention, and completeness of follow-up are the principal outcome measures. This study aims to establish that (a) the team can randomize 30 patients into this trial over the study period and (b) trial participants have > 90% adherence to receiving albumin (25%) or normal saline placebo boluses during SLED treatments, as assigned through randomization. For this outcome, each SLED treatment (rather than patient) will be the unit of analysis.

### Efficacy

Efficacy measures are to be assessed in an exploratory manner only. Efficacy outcomes will not be used to determine whether a subsequent, larger trial to assess efficacy outcomes is warranted. They will primarily be collected in order to identify any challenges with respect to data collection itself. Efficacy outcomes to be assessed by this pilot trial include (a) the “overall fluid removal” (i.e., percentage of target ultrafiltration achieved, calculated as the actual ultrafiltration volume divided by the target (ordered) ultrafiltration volume), (b) hemodynamic stability (i.e., initiation or increase in vasopressor dose(s) at the end of treatment, onset of MAP < 55 mmHg during treatment, absolute drop in MAP of ≥ 20 mmHg), (c) hospital length of stay, (d) ICU length of stay, (e) death at 1 year, and (f) dialysis dependence at 1 year. Efficacy measures (a) and (b) will be assessed at the level of SLED treatments while (c), (d), (e), and (f) will be analyzed at the patient level.

### Measurement

#### Albumin (25%) boluses

Participants randomized to this arm will receive 100 mL of intravenous albumin (25%) at the initiation of SLED and another 100 mL after 4 h of treatment (the half-way point of standard 8 h treatment). Both albumin and saline infusions will be administered over 15 min. The albumin boluses will be supplied through Canadian Blood Services.

#### Normal saline placebo boluses

Participants randomized to this arm of the trial will receive 100 mL of normal saline placebo at the initiation of SLED and another 100 mL after 4 h of treatment. The research pharmacy at The Ottawa Hospital will prepare the placebo saline boluses.

#### Co-interventions

As the development of severe or prolonged hypotension during treatment (for reasons related or unrelated to SLED) may necessitate urgent treatment, co-interventions will be permitted and tracked during the trial. This includes starting or increasing vasopressors and the administration of extra albumin (or other IV fluid) boluses during SLED, at the discretion of the treating physician.

### Ethical issues and trial registration

The pilot trial will be conducted in accordance with Health Canada’s Good Clinical Practice guidelines, the current Declaration of Helsinki, and the Tri-Council Policy State: Ethical Conduct for Research Involving Humans. The patients in this study are often not competent enough to consent to research due to their illness; many are intubated or are on respirator. Given this, we will have to seek consent for their participation from their designated substitute decision maker. Patients’ family who agree to participate in this study will provide a written informed consent by the research coordinator, included as Additional file 1. If during anytime of the study the patient regains competency, they will be provided with information, as is presented in the pamphlet included as Additional file 2, but will not be required to formally consent unless the intervention is ongoing (i.e., they are still receiving SLED). In most cases, patients are very unlikely to regain competency while still receiving SLED as it is only used as RRT when there is hemodynamic instability and patients tend to be severely ill and intubated. Patients can withdraw from the study at any time. Any data that were collected will then be discarded, destroyed, and not used in the study. All patients will be informed that they can withdraw from the study at any time. The study protocol, informed consent forms, and information pamphlet have been approved by the Ottawa Health Science Network Research Ethics Board. The trial is registered at the US National Institutes of Health (ClinicalTrials.gov) # NCT03665311.

Access to medical records and study data will be limited to authorized personnel listed on the study delegation log or permitted by the study agreement. Access to electronic data will be password protected and auditable, electronic data will be stored on a hospital network with firewall and security back-up measure in place, and paper copies of the study data will be stored securely in locked cabinets and in locked offices.

### Sample size and analytic plan

A total of 30 patients with AKI requiring treatment with SLED (by definition, Kidney Disease Improving Global Outcomes (KDIGO) stage 3 AKI) will be recruited and followed up for this study over the period of 7–14 months at the Ottawa Hospital’s intensive care unit. As this is a pilot study, we do not include a formal sample size calculation. We felt that including 15 patients in each group would inform us about the practical aspects of conducting a larger RCT with respect to study procedures, recruitment, and data collection. Of particular note, this includes determining the average number of SLED sessions received per patient (which will inform the analysis plan for efficacy outcomes for a larger study).

#### Baseline analyses

Baseline characteristics of patients in the two treatment arms will be assessed using frequency distributions and univariate descriptive statistics including measures of central tendency and dispersion.

#### Feasibility

For the feasibility outcome of recruitment, we will calculate the proportion (and 95% confidence interval) of all eligible SLED patients who are randomized into the trial. In addition, we will calculate the proportion of SLED patients that are eligible but declined to consent, eligible but not approached, and not eligible.

#### Efficacy

Efficacy outcomes in the pilot trial will be described using proportions for dichotomous variables. Continuous variables will be described using medians and interquartile ranges (IQRs). According to an assessment of the distribution of the efficacy outcomes data collected, appropriate statistical tests will be conducted to compare all efficacy outcomes between groups (with 95% confidence intervals). As mentioned, these outcomes will be underpowered for a meaningful comparison and are being conducted to help us design a multi-center pilot trial.

All analyses will be conducted using SAS® software Version 9.3 (Cary, NC, USA).

### Assignment of interventions and data management

The randomization process will consist of a computer-generated random listing of the treatment allocations. This random listing will be generated for four consecutive patients at a time, separately, at each of the two medical-surgical ICUs at The Ottawa Hospital (i.e., random assignment of individual patients to either treatment allocation *within* each block of four). This randomization will be accessed by the research pharmacist after a patient is enrolled as well as the assignment of intervention. After screening the patient for eligibility and obtaining informed consent, the study coordinator will provide the subject’s unique identification as well as a confirmation of consent and eligibility. The randomization and data management will be overseen by the Data Management Services (DMS) of the Methods Centre for the Clinical Epidemiology Unit of the Ottawa Hospital Research Institute. The DMS is responsible for designing, coding, implementing, and maintaining a secure web-based randomization system for the randomized controlled trial.

### Blinding

Only the research pharmacist responsible for preparing the albumin and placebo will access the computerized random allocation assignment. They will be the only member of the research team to record the allocation.

Blinding of the intervention is difficult since albumin (25%) must be stored in glass containers and is yellow in color while normal saline comes in infusion bags and is transparent. For this pilot trial, the blinding involves administering the placebo (normal saline) in similar glass bottles to those used for the albumin. Both the albumin fluid and placebo will be prepared in advance of administration by the research pharmacist, covered with opaque bags, and primed into opaque intravenous tubing. Furthermore, all members of the clinical and research team will be blinded except the research pharmacist.

### Progression criteria

We will apply the following criteria to make the decision that a definitive trial is worth pursuing, depending on funding and if we can successfully recruit more than 25 patients and enroll 15% of all eligible patients, of whom less than 10% have complications related to the intervention. This pilot trial is to test the study plan and find out whether enough participants will join a larger study and accept the study procedures. The results will be used as a guide for larger studies, and we plan to report our study findings in open-access peer-reviewed journals.

### Study measurement and patient safety

A trial management group involving the principal investigators (EC, SH), four co-investigators (LM, TR, AT, GK), and two study coordinators (IW, KM) will review, implement, and supervise all aspects of this pilot trial. All investigational product supplies in the study will be stored in a secure, safe place, under the responsibility of the investigators. The statistical analysis will be conducted by the investigators (EC and TR). The results will be disseminated via conferences and publication in an open-access publication and the trial registry; decision for publication lies with the investigators. The reporting of the results will follow the CONSORT statement- extension for Pilot and Feasibility Studies [20]. We will also continue to monitor and track any adverse events throughout this trial and report as necessary.

## Discussion

The results of the pilot trial will provide crucial data to the planning of a multi-center pilot randomized trial to determine if albumin (25%) boluses are as effective as normal saline boluses for improving hemodynamic stability and promoting fluid removal using SLED. A lower recruitment rate will help us to plan to add more sites or a longer recruitment period for the multi-center pilot trial. Protocol adherence and follow-up measurements will provide additional feasibility information to plan refinement of the final protocol as well as adjust sample size estimates for a larger trial. As well, the pattern of SLED use for enrolled patients (i.e., number of sessions per patient and determination of which sessions typically involve ultrafiltration/fluid removal) will help determine the optimal design of a larger trial with respect to whether randomization should occur at the patient or SLED treatment session level as well as the optimal number of treatments to consider for analysis.

If the results of a large multi-center trial showed that albumin boluses are effective in this setting then their wider-spread use might be warranted given mounting evidence that greater fluid overload in critically ill patients with AKI is associated with increased morbidity and mortality. If shown to be ineffective, the cost savings from decreased albumin use would be substantial.

### Limitations

The study design has certain limitations. Since this is a pilot trial, it will be underpowered to demonstrate efficacy; however, the potential efficacy data will allow planning for a larger trial. In addition, given the challenges of obtaining informed consent from critically ill patients, we conservatively assume a consent rate of only 20%. Even so, there should be a sufficient number of patients receiving SLED for AKI within less than 24 months.

### Trial status

This trial has received grant funding from The Department of Medicine, The Ottawa Hospital and the University of Ottawa in 2017, as well as obtained approval from the Ottawa Health Sciences Research Ethics Board on October 5, 2018. Patient recruitment will begin in April 2019.

## Additional files

Additional file 1: Consent form for AKI ICU patients to participate in the SAFER-SLED study. (DOCX 372 kb)

Additional file 2: Pamphlet information for AKI ICU patients who regain their competency to review. (DOCX 344 kb)

## Data Availability

Data from the trial will be made available following institutional policies.

## Abbreviations

AKI: Acute kidney injury
CRRT: Continuous renal replacement therapy
DMS: Data Management Services
ICU: Intensive care unit
IV: Intravenous
KDIGO: Kidney Disease Improving Global Outcomes
SAFER: Saline versus albumin for extracorporeal removal
SLED: Slow low-efficiency dialysis
TOH: The Ottawa Hospital
