# Supplementary figures and images for: Saline versus albumin fluid for extracorporeal removal with slow low-efficiency dialysis (SAFER-SLED): study protocol for a pilot trial
Source: Pilot Feasibility Stud. 2019 May 30;5:72. doi: 10.1186/s40814-019-0460-3 (PMC6542057; doi:10.1186/s40814-019-0460-3)

Additional file 1 Consent form for AKI ICU patients to participate in the SAFER SLED study.


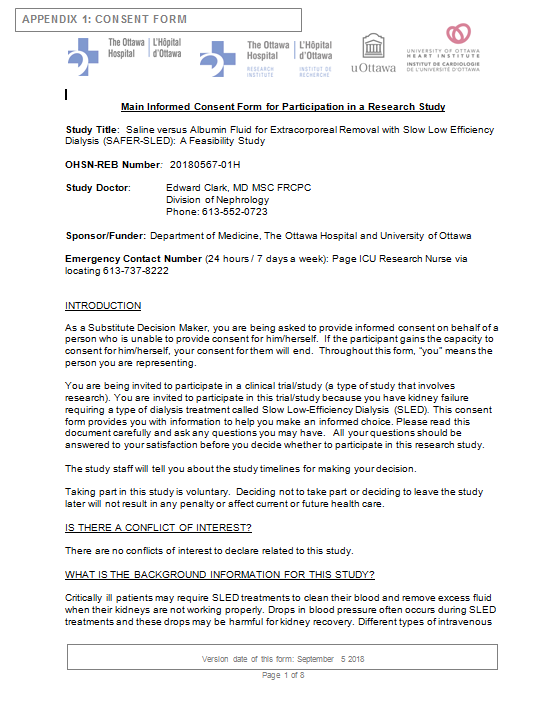


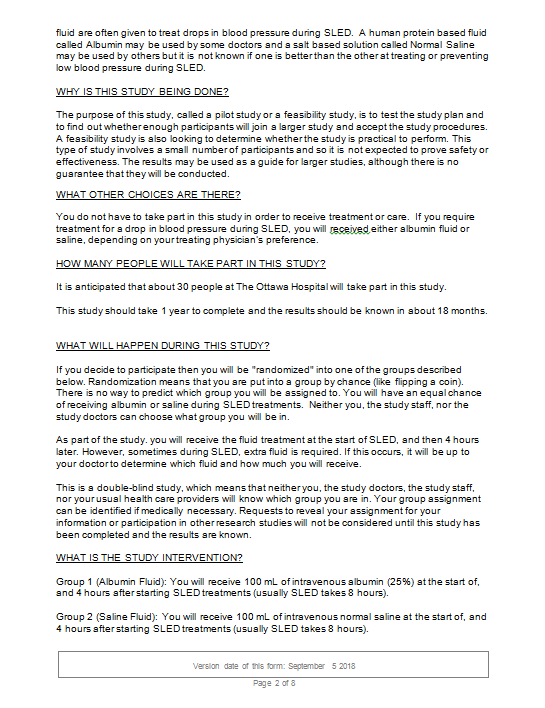


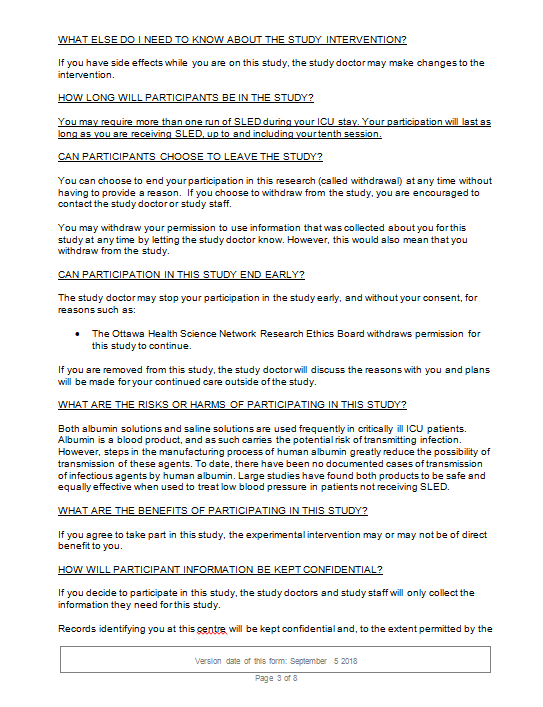


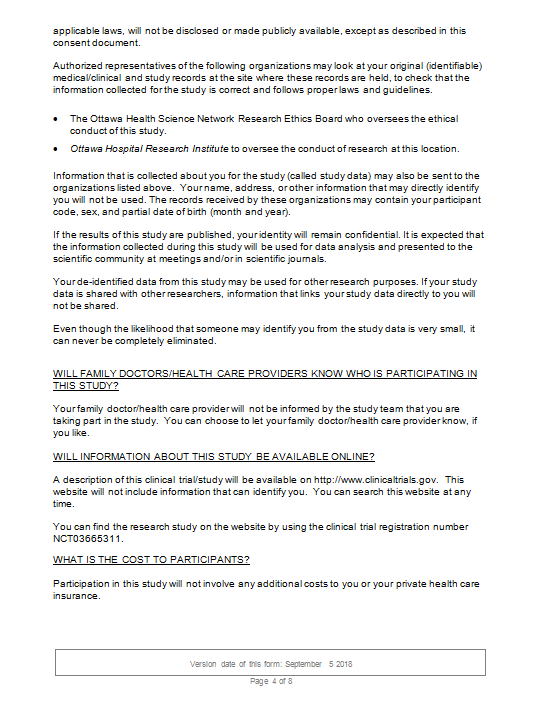


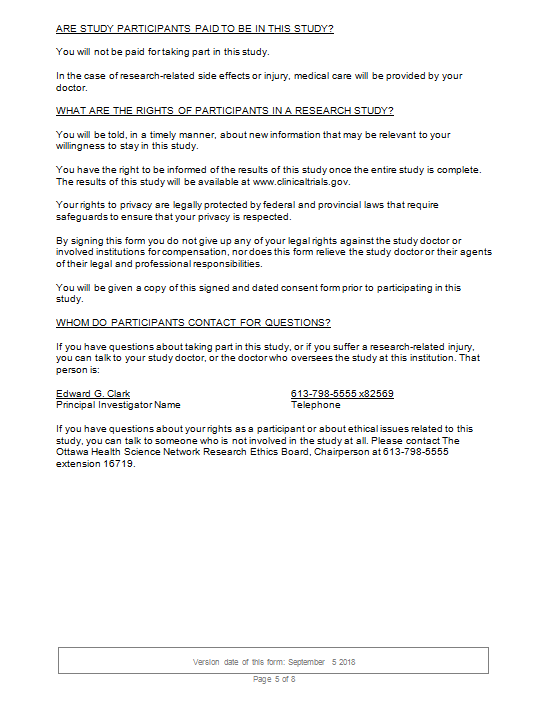


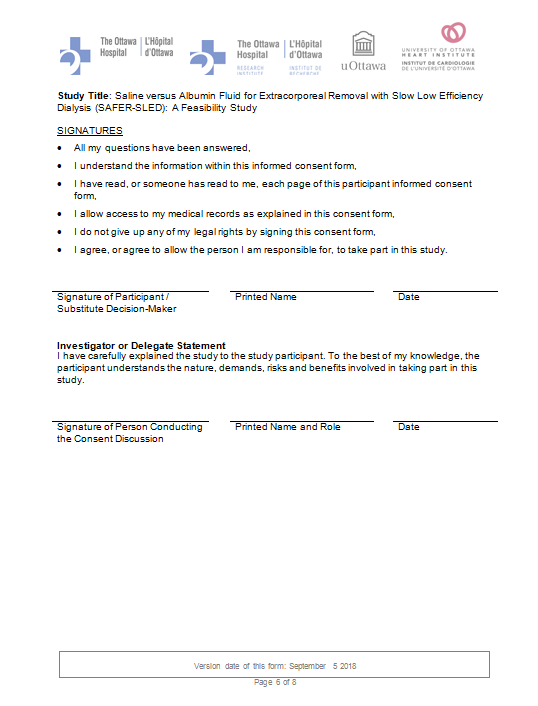


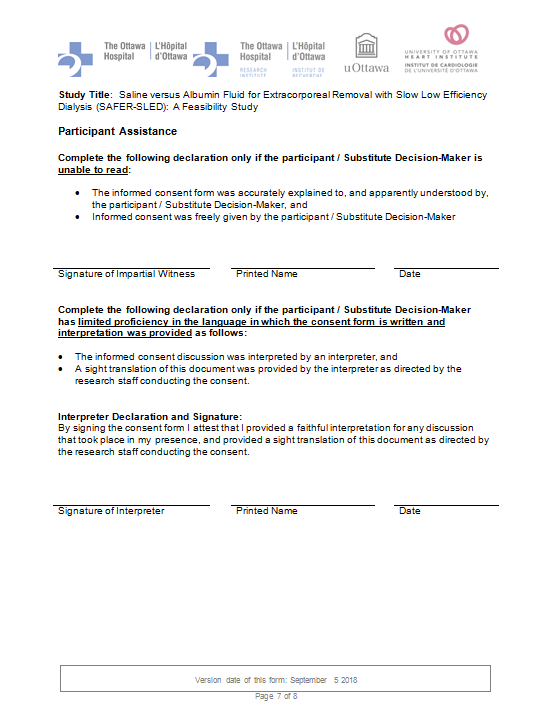


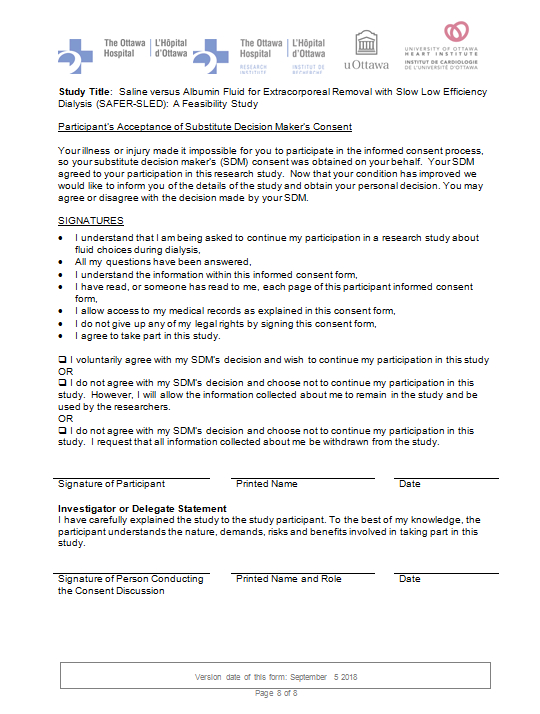

Supplement: Supplementary file 1 — Consent form for AKI ICU patients to participate in the SAFER-SLED study. (DOCX 372 kb) [file 40814_2019_460_MOESM1_ESM.docx]
