# Supplementary material for: Saline versus albumin fluid for extracorporeal removal with slow low-efficiency dialysis (SAFER-SLED): study protocol for a pilot trial
Source: Pilot Feasibility Stud. 2019 May 30;5:72. doi: 10.1186/s40814-019-0460-3 (PMC6542057; doi:10.1186/s40814-019-0460-3)

Additional file 2 Pamphlet information for AKI ICU patients who regain their competency to review.


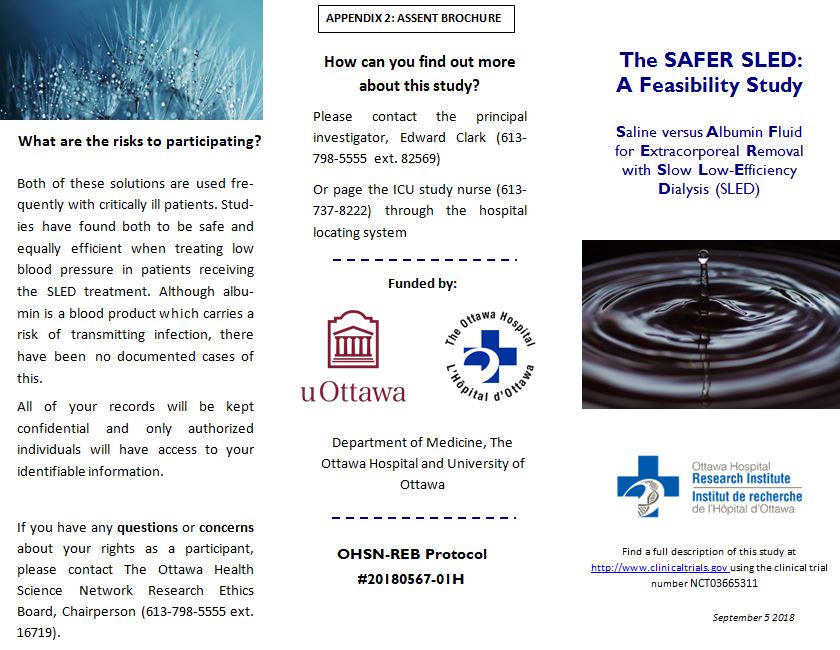


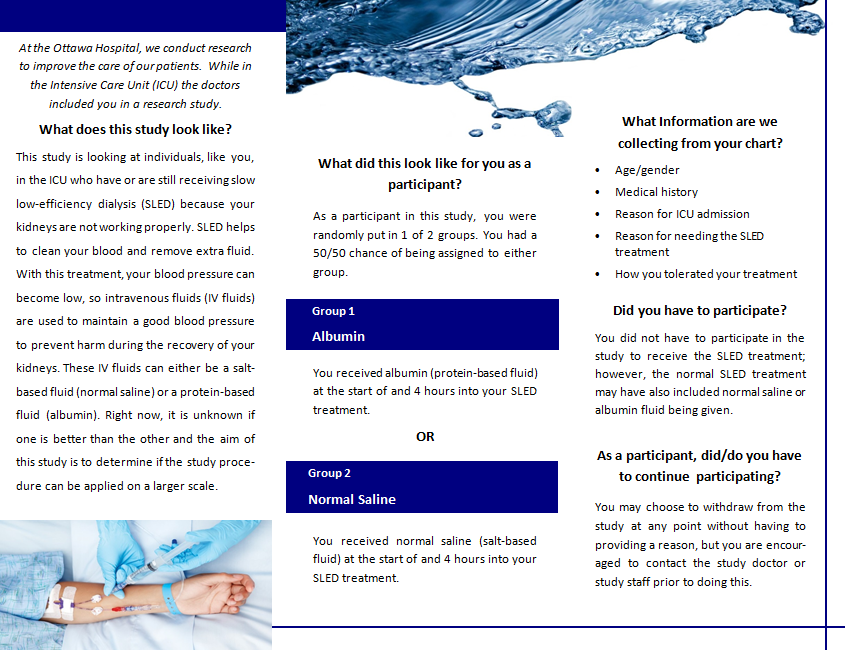

Supplement: Supplementary file 2 — Pamphlet information for AKI ICU patients who regain their competency to review. (DOCX 344 kb) [file 40814_2019_460_MOESM2_ESM.docx]
